# Learning-based physical models of room-temperature semiconductor detectors with reduced data

**DOI:** 10.1038/s41598-022-27125-7

**Published:** 2023-01-04

**Authors:** Srutarshi Banerjee, Miesher Rodrigues, Manuel Ballester, Alexander Hans Vija, Aggelos K. Katsaggelos

**Affiliations:** 1 Northwestern University, 2145 Sheridan Road, Evanston, IL 60208 USA; 2 Siemens Medical Solutions USA Inc, Hoffman Estates, IL 60192 USA

**Keywords:** Materials for devices, Electronic devices

## Abstract

Room-temperature semiconductor radiation detectors (RTSD) have broad applications in medical imaging, homeland security, astrophysics and others. RTSDs such as CdZnTe, CdTe are often pixelated, and characterization of these detectors at micron level can benefit 3-D event reconstruction at sub-pixel level. Material defects alongwith electron and hole charge transport properties need to be characterized which requires several experimental setups and is labor intensive. The current state-of-art approaches characterize each detector pixel, considering the detector in bulk. In this article, we propose a new microscopic learning-based physical models of RTSD based on limited data compared to what is dictated by the physical equations. Our learning models uses a physical charge transport considering trapping centers. Our models learn these material properties in an indirect manner from the measurable signals at the electrodes and/or free and/or trapped charges distributed in the RTSD for electron–hole charge pair injections in the material. Based on the amount of data used during training our physical model, our algorithm characterizes the detector for charge drifts, trapping, detrapping and recombination coefficients considering multiple trapping centers or as a single equivalent trapping center. The RTSD is segmented into voxels spatially, and in each voxel, the material properties are modeled as learnable parameters. Depending on the amount of data, our models can characterize the RTSD either completely or in an equivalent manner.

## Introduction

RTSDs are required for a large number of applications such as medical imaging, homeland security, astronomy and high energy physics^1–5^. These applications calls for high quality crystals at reasonable cost, with uniform and optimized charge transport properties - no polarization effect, excellent fabrication quality, high breakdown voltage, high drift velocity, high energy resolution and lowest possible defects (charge trapping centers). Over the last several decades, RTSDs such as CdTe, CdZnTe, HgI$_2$, TlBr have emerged as potential detectors. RTSDs are often used as compact radiation detection units with highly segmented pixelated anode patterns.

CdZnTe (CZT) is the leading RTSD of choice today. The performance and yield of high quality detector-grade materials are limited by presence of high concentrations of performance-limited defects which are randomly distributed. CZT suffers from major detrimental defects^6^ such as compositional inhomogeneity due to non-unity segregation coefficient of Zn^7^, presence of high concentration of secondary phases, Te inclusions and sub-grain boundaries/dislocation walls in high concentration in CZT array. These defects act as trapping centers, hindering localized charge transport and imposes spatial non-uniformity in charge transport properties, thereby adversely affecting the detector performance^8–13^.

The efforts to characterize these detectors has been done over last several years. For instance, using thermoelectric emission spectroscopy (TES) and thermally stimulated conductivity (TSC) measurements, the thermal ionization energies of the electron and hole traps were measured^14^. Determination of the trap lifetime was done using a microwave cavity perturbation method in CdZnTe and HgI$_{2}$^15^. In another work, the electron and hole traps of CdZnTe samples were radially irradiated with 5 MeV focused proton beam to generate electron–hole pairs and fill traps, which were released later by thermal re-emission. Electron and hole traps were distinguished by excitation near the vicinity of the appropriate electrodes^16^. Deep trap levels in CdZnTe were characterized by analyzing simultaneous multiple peaks on TSC measurements^17^. 9 defect levels and irradiation-induced variations of these levels were observed on CdZnTe:Al using TSC measurements^18^. The average hole trapping time $\tau _{h}$ was derived using statistical model of charge collection efficiency based on known electron average trapping time^19^. Average hole de-trapping time $\tau _{dh}$ was extracted by comparing the measured and simulated signals for holes as measured in the cathode^20–22^. The effects of deep-level defects on the carrier mobility in CZT have been studied^23^. 13 trap levels in Indium doped CZT crystal had been shown^24^. Imperfections due to mechanical damage or adsorbed chemical species trap charges or increase leakage current. Using pulsed laser microwave cavity perturbation method selectively at the surface and in the bulk region of CZT, these defects has been characterized^25^. The influence of type of metal contacts and deposition techniques on the recombination and trapping defects at the metal-semiconductor interface has also been studied^26^. The uniformity of high flux CdZnTe has also been characterized^13^. In literature, the defects and charge transport properties of electrons and holes are measured using classical approaches considering homogeneous behavior over the detector. This requires cumbersome multiple experiments and technical know how. However, the homogeneity and repeatability of the defects and charge transport properties within a detector and across multiple detectors are unknown. However, achieving high energy resolution below $1.0\%$ at 662 keV and sub-millimeter position detection accuracy depends on in-depth characterization of the RTSDs. Such characterization of RTSD will also aid in developing improved reconstruction algorithms. However, this approach traditionally is hugely time consuming, requires numerous sophisticated experiments and skilled manpower. Thus, for RTSD arrays, precise characterization of each detector spatially and temporally is vital.

A Machine Learning based approach is used to address this problem. Machine learning has been tremendously popular in the last few years with several novel works in different fields. Recent focus on applying machine learning to materials, physics based systems, drug discovery is gaining momentum. Integrating Physics-based Modeling and Machine Learning is becoming more popular over the years^27,28^. The overall objectives of such approaches are to develop inverse models, improve predictions beyond state-of-art physical models, model parameterization, partial differential equations (PDEs) solutions, discover symbolic governing equations, and others. Solving problems in physics governed by PDEs using Neural Networks has been done^29,30^. These models are typically based on physics guided loss function, initialization, architecture, hybrid model of physics with Deep Learning (DL) and other approaches. For example, the two-dimensional wave equation is modeled as a Recurrent Neural Network^31^. DeepONets^32^ have been demonstrated as a powerful tool to learn nonlinear operators in a supervised data-driven manner. The 2D Poisson Equation has been solved with a Physics Informed Neural Networks^33^. In most of these physics based machine learning approaches, relatively simpler PDEs have been solved. However, the charge transport in a RTSD has multitude of coupled PDEs^34^ involving charge drift, trapping, detrapping and recombination, which has been addressed only in one of our previous works^35^ using a physics-inspired learning model.

Our approach follows the principle of physics-guided design architecture. Our previous work^35^ focused on characterizing radiation detectors using learning-based approaches, where adequate data dictated by physical equations of electron and hole charge transport in the RTSD. These are the signals at the electrodes, free and trapped charges in the bulk of the material, which were used to train and test the learning-based model. In our previous work, the physical charge transport equations were used in the model architecture with input parameter as the injected electron–hole charge pairs at different voxels and output parameters as the free and trapped charges, alongwith signals at the electrodes. All the output parameters were used for training and testing the model, which we refer as the model with adequate data. However, in reality, although the signals are readily available from the electrodes, obtaining data such as free and trapped charges in the bulk of the RTSD with several trapping centers is cumbersome.

In this paper, our main contributions has been to develop physics-inspired learning models derived from the physical charge transport equations for both electrons and holes for a RTSD based on fewer data than what is dictated by the physics of charge transport in RTSDs. Depending on the amount of data, this result in models which characterizes the material either completely for multiple trapping centers or in a single equivalent trapping center. Adequate data can be obtained using numerous hardware experiments, which is time consuming, and requires skilled manpower. This work proposes physics-based models to characterize the RTSD with fewer simulation data. This would be hugely beneficial for characterizing the detector in large scale and implementing in different applications—medical imaging, security, astronomy and others, without much additional effort and experimental setups. Depending on the amount of data compared to what is desired by physical equations, the detector can be characterized either precisely based on the numerous trapping centers or as a single equivalent trapping center. As in our previous work^35^, the detector is spatially discretized into voxels. The physical charge transport equations are incorporated into the architecture of the model, in each voxels of the model. A conventional CNN or RNN model typically has millions of trainable weights. However, incorporating the physical charge transport equations into the architecture of the model results in fewer trainable weights and requires less training data, as the amount of training data is proportional to the number of trainable parameters. The trained weights are one-to-one related with the RTSD material properties. We use simulated data for training our learning-based physical model of charge transport in RTSD.

Our learning-based physical models is an attempt to solve the vital problems currently plaguing the characterization of radiation detectors—(a) Characterization of detector material properties at finer resolution (at micron scale) in a fast and efficient way, (b) Characterization of defects in the material depending on the amount of data, and (c) Characterization of the material precisely based on multiple trapping centers or as a single equivalent trapping center.

## Methods

In this section, we present the classical approach for detector modeling, learning-based full model of the detector and our learning-based physical model of detectors with reduced data. Our approach of developing the learning-based physical models based on reduced data has been progressively developed with reduced amount of data during training the models, thereby reducing the amount of learnable parameters (and hence the material properties) in the model as shown in the subsequent sections of the paper.

### Classical approach for detector modeling

Electrons and holes transport properties play a vital role in selecting the RTSD. Shockley-Read-Hall (SRH) Theory^36,37^ governs the trapping, detrapping and recombination during charge transport. Rodrigues et al.^38^ measured the detailed properties of these detectors using the charge transport and charge continuity equations with multiple electron and hole defect levels—coupled with Poisson’s equation^39–43^. In the RTSD, the electron–hole pairs are created when high energy photons interact with the material. Subsequently in the RTSD, the free charges drift towards the respective electrodes, along with trapping, de-trapping in the defect levels and recombination of free charges in the bulk of the material^44^. The temporal dynamics of free electrons and holes following the SRH model is affected by the trapping energy states in the bandgap^45^. For free hole concentration (*p*), with trapping and detrapping lifetimes $\tau _{Ti}$ and $\tau _{Di}$ respectively for *i*th trap, and $p_{ti}$ as the concentration of holes in the *i*th trap, the following equation can be written^45^,

1 $$\begin{aligned} \frac{dp}{dt} =\sum _{i} \left( -\frac{p}{\tau _{Ti}}+\frac{p_{ti}}{\tau _{Di}} \right) \end{aligned}$$

**Figure 1:**
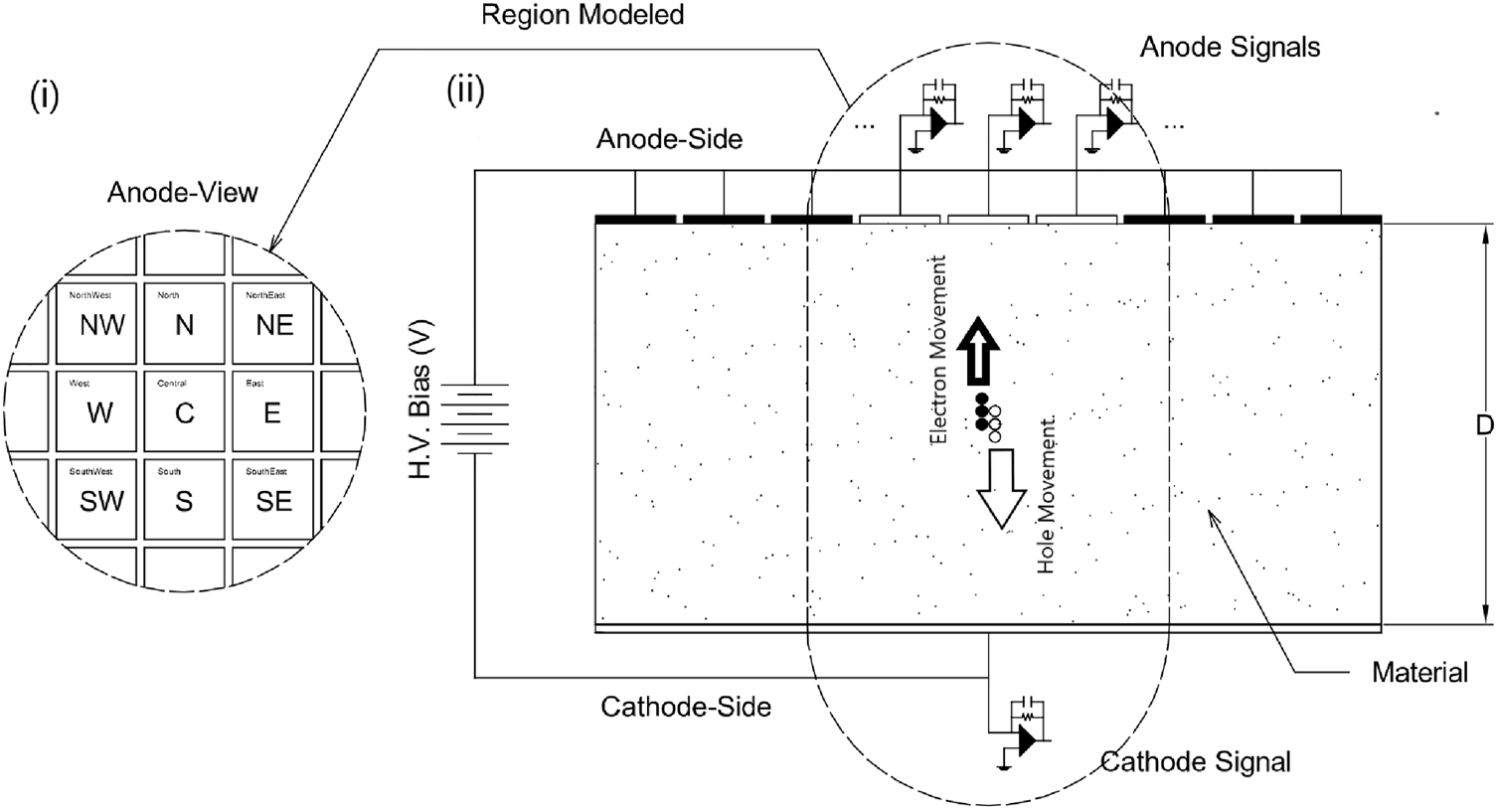
Detector Configuration (i) Pixelated anode pattern with 9 pixels as the regions of interest, (ii) Detector configuration with pixelated anode on top, CZT in the middle and, cathode at the bottom. Figure adapted with permission^35^.

The trapping and detrapping lifetimes dictate whether the defects induce short term or long term trapping of charges in the detector. Considering the low probability of transition of charges between the trapping centers, the occurrence of such process^46^ is neglected in Eq. (1). The equations governing the transport, trapping and detrapping, and diffusion of electrons and holes is detailed^35^. Signals collected at the electrodes arise due to the movement of charges^47–50^. The detector setup is shown in Fig. 1 with the 9 grid electrodes on the anode side—north–west (NW), north (N), north–east (NE), west (W), center (C), east (E), south–west (SW), south (S), south–east (SE), and, 1 single large cathode electrode (CAT).

The simulated data for training the proposed learning model has been generated using the classical physical equations^34,35^. A MATLAB code was developed for describing the charge transport equations in the detector, by defining the transport, trapping, de-trapping and lifetimes of electrons and holes which are $\mu _{e}$, $\mu _{h}$, $\tau _{eT}$, $\tau _{eD}$, $\tau _{hT1}$, $\tau _{hD1}$, $\tau _{hT2}$, $\tau _{hD2}$, $\tau _{e}$, and $\tau _{h}$ respectively as the fixed pre-defined parameters, with electric field along the material^35^. As in our previous work^35^, the classical model was created for a discretized (voxelized) RTSD, with charge input at any voxel. At each time step, the signals are collected at the cathode and pixelated anodes. The free and trapped charges in different voxels are also computed at each time step. The time steps and total number of time steps is defined *a priori*. The input for training the learning-based model consists of electron–hole pairs injected at known voxel positions. The signals, alongwith free and trapped charges in different voxels of the classical model over different time steps are the output of the learning-based model^35^.

### Learning-based full model of detector

**Figure 2:**
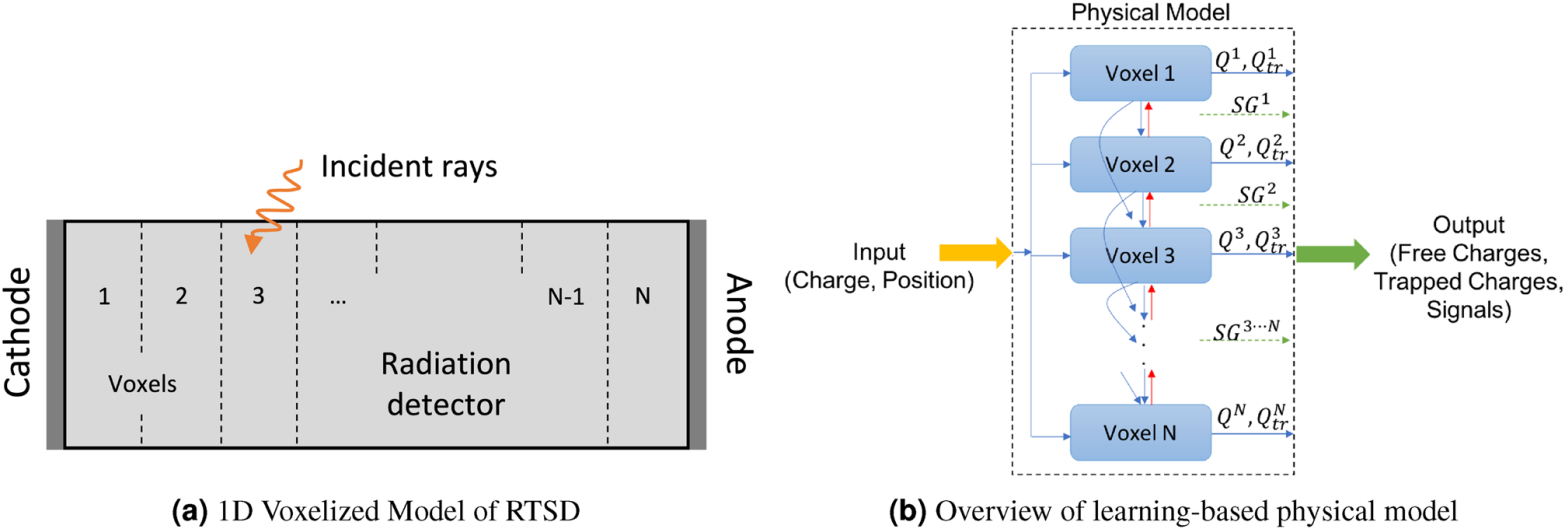
RTSD model with (**a**) 1D voxels and electrodes at the either ends, and (**b**) Overview of the learning-based physical model of RTSD. The input to the model is the electron–hole charge and position. The learning-based physical model consists of trainable weights in each voxel. The charges move dynamically from one voxel to another based on the Electric Field and polarity (blue for electrons, and red for holes). The free and trapped charges in each voxel alongwith the signals generated during movement of charges are used as output of the model. Cathode and Anode is on outer ends of Voxel 1 and N respectively.

**Figure 3:**
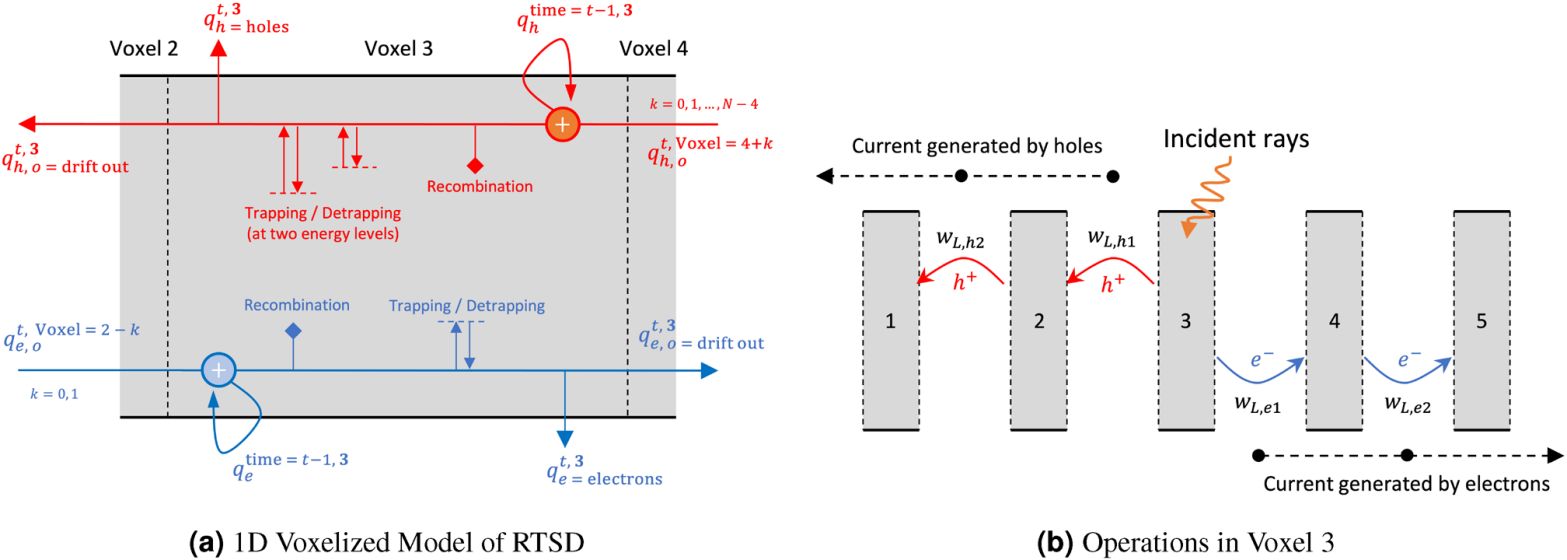
(**a**) Operations at voxel 3 at a particular time t. The electron transport from cathode to anode (left to right) is shown in the bottom half of this figure, while the hole transport in the opposite direction is shown in the top half of the figure. Figure adapted with permission^35^, and (**b**) Current generation in the RTSD. For simplification, we show the 1D model with only five voxels. When the $\gamma$-rays hit voxel 3, an electron–hole pair is produced. Charge transfer from one voxel to another induces a potential difference *w* at the electrodes. The product of transferred charge with *w* gives the generated current at the electrodes. Figure adapted with permission^35^.

**Figure 4:**
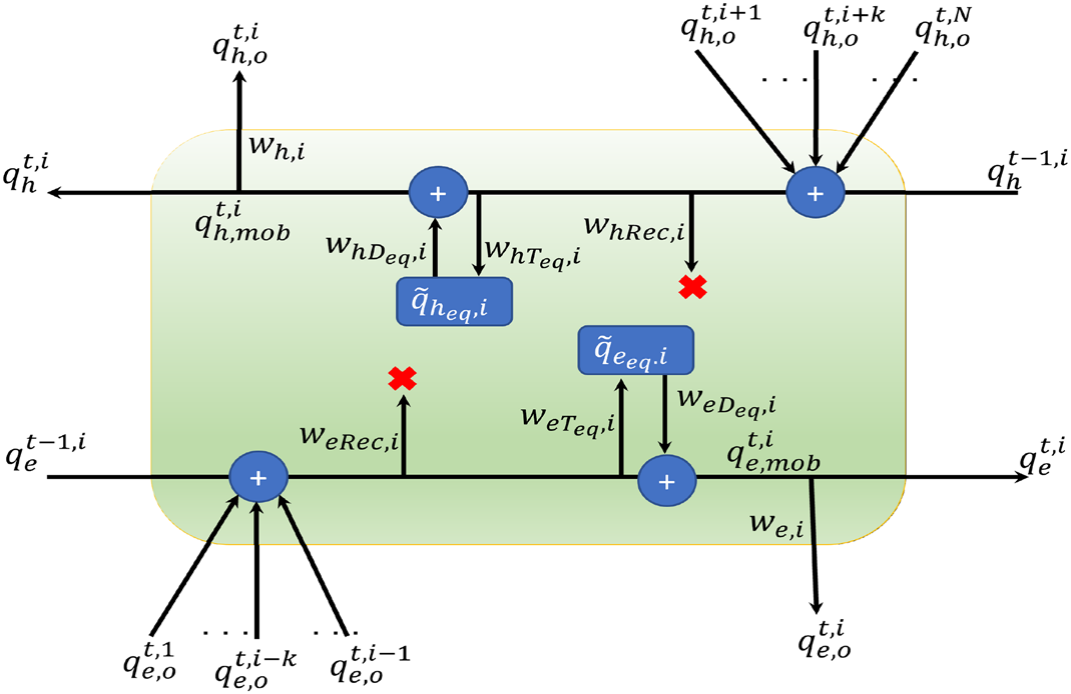
Equivalent Operations in voxel *i* at time *t*.

We subdivide the RTSD into *N* voxels, (*N* = number of subdivisions in the material in any dimension). In each of the discretized voxels, the material properties such as $\mu _{e,h,i}$, $\tau _{eT,i}$, $\tau _{eD,i}$, $\tau _{hT_{1},i}$, $\tau _{hD_{1},i}$, $\tau _{hT_{2},i}$, $\tau _{hD_{2},i}$, $\tau _{e,i}$, and $\tau _{h,i}$ are defined, which refer to the drift coefficients for electrons and holes, electron trapping lifetime, electron detrapping lifetime, hole trapping 1 lifetime, hole detrapping 1 lifetime, hole trapping 2 lifetime, hole detrapping 2 lifetime, electron lifetime and hole lifetime respectively in *i*th voxel^35^. The model therefore allows the determination of the unknown material properties with higher spatial resolution than the bulk of the RTSD. For each of these coefficients (referred here as $\tau$ in general), we compute the number of charged particles (electrons or holes) remaining in that particular level as $N_{left} = N_{0}e^{-{t}/\tau }$, where $N_{0}$ and $N_{left}$ are the number of charged particles at a particular level at $t=0$ and at time *t* respectively^35^. We use these charges in our model. For a particular material property $\tau$, we can find out the fraction of charges remaining in that energy level. A voxelized representation of the detector in 1D is shown in Fig. 2a with the electrodes at either end - anode on the right and cathode on the left. The high energy radiation (for example Gamma rays or X-rays) can interact in any position of the RTSD. The learning-based model is a recurrent network structure over time with the input to the model as the injection positions of the electron–hole pairs with the magnitude of the injected charges normalized to 1 as shown in Fig. 2b. Each voxel consists of discretized material properties as the trainable weights. As charges drift under the influence of Electric Field, the electrons moves towards the anode (blue arrows in Fig. 2b) and the holes moves towards the cathode (red arrows in Fig. 2b). The operations in each voxel can be either that of the full model as shown in Fig. 3a or as an equivalent model as in Fig. 4. Each voxel consists of free and trapped electron and hole charges at each time instant. The movement of charges between the different voxels gives rise to signals at the electrodes. The output of the model are the signals from the electrodes, free and trapped electron and hole charges in the voxels over time. Based on the electron–hole pair input to this model, the outputs (signals, free and trapped charges) are computed over time^35^.

Figure 3a shows the operations taking place in voxel *i* at time *t*. At any time $t-1$, the charge in voxel *i* in free state is $q^{t-1,i}_h$ for holes and $q^{t-1,i}_e$ for electrons. Under the influence of Electric Field from Anode to the Cathode, holes drift at time *t* from other voxels $i+1,\ldots , i+k,\ldots , N$, to voxel i, with charges $q^{t,i+1}_{h,o},\ldots , q^{t,i+k}_{h,o},\ldots , q^{t,N}_{h,o}$ respectively. These charges are added onto the existing charge to form the total charge due to holes in voxel *i* at time *t*. Some of this net hole charges recombine with the intrinsic electron concentration in the bulk of the material. The presence of trapped hole centers in the RTSD traps some of the holes and detraps holes back as excess hole concentration over bulk. In Fig. 3a, we show 2 trapping centers for holes and 1 trapping center for electrons which resemblances a typical CZT detector^14,34^. However, this modeling approach can be used for other RTSDs as well with several trapping centers for electrons and holes, depending on its material properties. A fraction of holes drift out of voxel *i*, while the remaining holes are left behind in voxel *i* at time *t*. Similar operations are repeated for electrons, as shown in bottom half of Fig. 3a. This approach is as per our previous work^35^.

Figure 3b shows the model with the detector discretized into 5 voxels (for illustration). The high energy rays are incident on voxel 3 creating electron–hole pairs in that voxel. The electrons drift towards the anode (right of voxel 5), while the holes drift towards the cathode (left of voxel 1). While drifting from one voxel to another, the electron charges are multiplied by difference of induced potentials, to generate electrical current from movement of electrons ($signal_{electrons}$) as per the Schokley-Ramo Theorem^48^. Similar phenomena occur to generate electrical current from movement of holes ($signal_{holes}$). The induced potential differences due to the motion of charges are fixed based on the geometry and pre-computed in this model^35^.

Additionally, we consider the applied bias voltage to the electrodes to be fixed at $V_i$ and $V_f$. In general, the voltage can vary in any manner within the RTSD depending on the defects within the material. Here we consider the voltage at each voxel of the detector to be a linearly increasing function from cathode to the anode. An error term $error_{voltage}$, is formulated as the difference between the voltages at a particular voxel obtained from successive linear segments, as detailed in our previous work^35^.

The learning-based model is trained with the data simulated in MATLAB. The input is the voxel position and magnitude of the injected electron–hole pair. The signals obtained at the electrodes along with the electron and hole charges (free and trapped) in each of the voxels over time are the output of this learning-based model. The overall loss function is computed as the sum of the squared errors between the signals and charges in the voxels compared to the ground truth signals along with the $error^{2}_{voltage}$. We consider CZT detector with 2 trapping centers for holes and 1 trapping center for electrons^14,34^ as shown in Eq. (2). In the loss function, the subscript *gt* refers to the ground truth data for the particular parameter generated in MATLAB, while the subscript *L* refers to the data generated by the learning based model as detailed in our previous paper^35^.

2 $$\begin{aligned} LF = & {} k[(signal_{gt}-signal_{L})^2 + error^2_{voltage}] + l[(q_{e,gt}-q_{e,L})^2 + (q_{eT_{1},gt}-q_{eT_{1},L})^2] + n[(q_{h,gt}-q_{h,L})^2 + (q_{hT_{1},gt}-q_{hT_{1},L})^2 + (q_{hT_{2},gt}-q_{hT_{2},L})^2].\end{aligned}$$

### Learning-based physical models of detector with reduced data

The learning-based full model of the detector uses a loss function taking into consideration the complete data as required by the classical physical equations—signals, voltage distribution in the material, free and trapped charges in the different trapping centers for both electrons and holes. In the real world, each of these data must be obtained from experimental hardware setups with the detector, which not only requires costly equipments, but also skilled manpower and time. In order to address this issue, we propose learning-based models to learn from fewer data than in the full model (which is dictated by the classical physical model). We explore the models by training with fewer data than what is dictated by the classical physical equations, step-wise removing small fraction of relevant data from the full learning-model (such as charge trapped and trapping centers) and evaluate the performance of the learning-model. In the end, we remove a significant portion of the data from the full model and use only the signals for training the physical model. Our models have been developed keeping in mind what can be measured with the hardware setups and labor required to generate these data.

#### Physical Model-1

The Physical Model-1 has been developed using signals at the electrodes, voltage distribution along the detector, free electron charges and, free and trapped hole charges in one trap center as shown in Eq. (3). We use hole charges corresponding to one trapping center and no electron charges for its trapping center for CZT detector with 2 trapping centers for holes and 1 trapping center for electrons. Our model as shown in Figs. 2 and 3 can be trained using charges corresponding to any one of the hole trapping center. However, for illustration purposes, we have used data from hole trapping center 1. For any other material with $NT_h$ trapping centers for holes and $NT_e$ trapping centers for electrons, we would use the data corresponding to $NT_e - 1$ trapped charges for electrons and $NT_h - 1$ trapped charges for holes.

3 $$\begin{aligned} LF_{1} = k[(signal_{gt}-signal_{L})^2 + error^2_{voltage}] + l[(q_{e,gt}-q_{e,L})^2] + n[(q_{h,gt}-q_{h,L})^2 + (q_{hT_{1},gt}-q_{hT_{1},L})^2]. \end{aligned}$$

The learning-based model uses the same hyperparameters *k*, *l*, *n* in the loss function in Eq. (2) which was derived through optimization in our earlier work^35^.

#### Physical Model-2

In Physical Model-1, we observed from our simulation experiments that the hyperparameters *k*, *l*, *n* in the loss function are biased heavily towards the free and trapped charges, and hence the hyperparameters *l*, *n* are much higher than *k*. Thus, the Physical Model-2 has been developed considering only the free electron charges and, free and trapped hole charges, corresponding to hole charges in only one trapping center—in this case using hole charges in hole trapping center—1 (for illustration purposes). The loss function ($LF_2$) is then defined in Eq. (4),

4 $$\begin{aligned} LF_{2} = l[(q_{e,gt}-q_{e,L})^2] + n[(q_{h,gt}-q_{h,L})^2 + (q_{hT_{1},gt}-q_{hT_{1},L})^2]. \end{aligned}$$

The model does not use the signals and the voltage distribution in bulk of the detector, as well as the hole charges in the trapping center 2 of the CZT detector to characterize the material. In general, for $NT_h$ trapping centers for holes and $NT_e$ trapping centers for electrons, we can use the free hole and electron charges, as well as, electron and hole trapped charges for $NT_e - 1$ and $NT_h - 1$ trapping centers respectively in training the model.

#### Physical Model-3

In the Physical Model-3, we further reduce the dependency on any of the trapped hole charges which were used in the Physical Model-2. This results in a model which can characterize the trapping centers in an equivalent manner. The equivalent trapping and detrapping lifetimes are the equivalent contribution of several trapping and detrapping lifetimes in the detector which contributes to the dynamics of charge motion in the detector as shown in Eq. (1). The properties of the physical detector can be attributed as defect-free properties in addition to equivalent defects in the material. The detector has inherent properties such as transport of charges (electrons and holes) in bulk of the material alongwith recombination of charges which form the defect free model. On the other hand, the defects (equivalent) in the model are contributed due to trapping and detrapping of charges at the trapping centers within the detector. The presence of multiple trapping and detrapping levels can be converted to equivalent trapping and detrapping levels. In such a scenario, for 2 hole trapping levels of CZT with trapping lifetimes $\tau _{1}$ and $\tau _{2}$, the equivalent trapping lifetime is given in Eq. (5),

5 $$\begin{aligned} \frac{1}{\tau _{eq}} = \frac{1}{\tau _{1}} + \frac{1}{\tau _{2}}. \end{aligned}$$

Considering the probability of trapping 1 level as $p_{\tau _{1}}$ and detrapping 1 lifetime as $\tau _{dt_{1}}$, alongwith probability of trapping 2 level as $p_{\tau _{2}}$ and detrapping 2 lifetime as $\tau _{dt_{2}}$, we can calculate the equivalent detrapping lifetime as in Eq. (6),

6 $$\begin{aligned} \frac{p_{\tau _{eq}}}{\tau _{dt,eq}} = \frac{p_{\tau _{1}}}{\tau _{dt_{1}}} + \frac{p_{\tau _{2}}}{\tau _{dt_{2}}}. \end{aligned}$$

The physical model-3 is designed as a combination of defect-free model and model with equivalent defects. The equivalent computations in a voxel *i* is shown in Fig. 4. The equivalent trapping and detrapping weights are $w_{hT_{eq},i}$ and $w_{hD_{eq},i}$ for holes, and similarly for electrons, the corresponding trapping and detrapping weights are $w_{eT_{eq},i}$ and $w_{eD_{eq},i}$. The charges in equivalent trap center is $\tilde{q}_{h_{eq},i}$ and $\tilde{q}_{e_{eq},i}$ for holes and electrons respectively. The loss function consists of only the free electron and hole charges is used to train the model, as shown in Eq. (7),

7 $$\begin{aligned} LF_{3} = l[(q_{e,gt}-q_{e,L})^2] + n[(q_{h,gt}-q_{h,L})^2]. \end{aligned}$$

#### Physical Model-4

In Physical Model-4, we use only the signals generated at the cathode and anodes due to motion of the charged particles to train. Typically, the signals are generated at the electrodes by the superposition of signals generated individually due to transport of electrons and transport of holes. However, in Physical Model-4, we separate out the signals generated due to the transport of electrons from the signals generated due to the transport of holes and use them in training the model. The loss function for training the physical model-4 is shown in Eq. (8),

8 $$\begin{aligned} LF_{4} = [(sg_{e,gt}-sg_{e,L})^2] + [(sg_{h,gt}-sg_{h,L})^2]. \end{aligned}$$

It is also observed from our simulation experiments that by using the loss function in Eq. (8) leads to the trained model weights which fail to converge to the ground truth solution. The solution converges to a local minimum which is different from global minimum, and hence the trained weights differ from the ground truth weights. We use a Total Variation (T.V.) regularization on the different weights of the model corresponding to the trapping, detrapping and recombination of electrons and holes to converge the learned solution to the global minimum solution as observed in our simulation experiments. Similar to Physical Model-3, the multiple trapping and detrapping hole coefficients in actual material is learned in the Physical Model-4 as a single equivalent trapping and detrapping weights as well. The loss function gets modified to $LF_{4,m}$ as shown in Eq. (9),

9 $$\begin{aligned} LF_{4,m}= & {} [(sg_{e,gt}-sg_{e,L})^2] + \lambda _{1} {\Vert \nabla w_{eT_{eq}} + \nabla w_{eD_{eq}} + \nabla w_{e,Rec}\Vert }_2 + [(sg_{h,gt}-sg_{h,L})^2] + \lambda _{2} {\Vert \nabla w_{hT_{eq}} + \nabla w_{hD_{eq}} + \nabla w_{h,Rec}\Vert }_2. \end{aligned}$$

The T.V. regularization ensures smoothness in the trained weights of the model. The optimal values of $\lambda _{1}$ and $\lambda _{2}$ are determined through simulation experiments by finding the minimum error between the ground truth weights and the trained weights.

#### Implementation details

The learning-based physical models of RTSD with reduced data is trained with synthetic data by considering the classical model in MATLAB as in^35^. The scarcity of experimental data in literature for signals and charge distribution in bulk of the RTSDs is the major reason for developing an algorithm for generating simulated data for training and testing the different learning-based physical models. In these learning-based physical models, the input to the model are the injection positions and magnitude of the generated electron–hole pair charges. The magnitude of the injected charges are normalized to 1. The output from the model are the free and trapped charges in the voxels, alongwith the signals at the electrodes. The complete data as dictated by the physical equations for the RTSD is first generated using all the known phenomena such as drift, trapping, detrapping and recombination of electrons and holes. Subsequently, limited data is chosen out of these complete data in order to train the learning based models with complete physical properties for multiple trapping centers or a single equivalent trapping center, depending on the data.

The model weights are initialized during the start of the training process to its initial values. The models are trained over several epochs by computing the loss function based on the output corresponding to each input injections for the different reduced models. The model is a recurrent network structure over time, and hence Backpropagation through Time (BPTT)^51,52^ is used to compute the gradients of the loss with respect to the trainable weights in the model. The weights are updated based on a stochastic gradient descent method - ADAM optimization^53^. The learning rate of $5 \times 10^{-4}$ is used alongwith 2 momentum terms set as $\beta _{1}=0.9$ and $\beta _{2}=0.999$. The optimization reduces the loss function over epochs and the weights are trained. Once the model is trained, the weights of the model converges to the ground truth detector parameters used to generate the data in MATLAB. Our model has been developed using the popular machine learning Tensorflow library^54^ in Python in eager execution mode.

The trained weights for the different Physical Models has been evaluated by computing an error metric for each of the weights. For example, for electron trapping weight ($w_{eT}$) with $w_{eT,gt}$ and $w_{eT,lr}$ as the ground truth and trained weights respectively, the error is expressed as,

$$\begin{aligned} Err(w_{eT}) = \sqrt{ \frac{1}{N_{fin}-N_{inj} + 1} \sum _{i=N_{inj}}^{N_{fin}}\left\{ \frac{w_{eT,lr,i} - w_{eT,gt,i}}{w_{eT,gt,i}} \right\} ^2}. \end{aligned}$$

The error is computed over the injection positions of the electrons/holes and the number of voxels over which the weight gets trained over the epochs. The difference between the trained weights and ground truth weights for the trained region is normalized by the ground truth weights, to take into account the different ranges of weights in the model and put equal emphasis on the different model weights. For multiple injections of electron–hole pair, when the model weights are not trained in a contiguous manner, only the voxels where the weights are trained are taken into account in order to compute this error metric. For characterization purposes in this paper, more emphasis is placed on the RTSD properties in the bulk of the material than at the ends. The mean error (*Err*(*Mean*)) is computed as the arithmetic mean of these individual weights. The relative error, expressed as

$$\begin{aligned} Err2(w_{eT}) = \frac{1}{N_{fin}-N_{inj} + 1} \sum _{i=N_{inj}}^{N_{fin}}\left|\frac{w_{eT,lr,i} - w_{eT,gt,i}}{w_{eT,gt,i}}\right|\times 100 \end{aligned}$$

for each of the trained weight are also shown for electrons and holes for the four different physical models. For electron and hole weights, the mean of the different electron and hole weights are computed as the relative error metric.

## Results

In this section, we present the simulation experimental results from the different physical models which have been presented in the previous section.

### Numerical experiments with Physical Model-1

**Figure 5:**
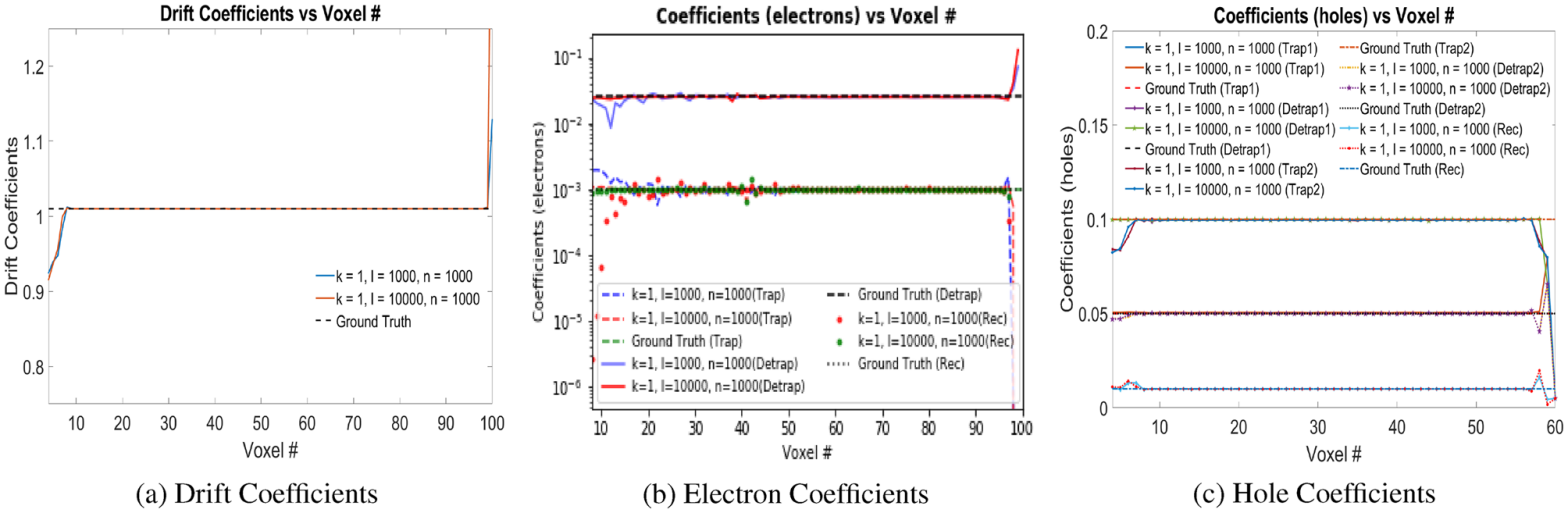
(**a**) Drift coefficients ($\mu _e$), (**b**) electron coefficients ($w_{eT}$, $w_{eD}$, $w_{eRec}$), and, (**c**) hole coefficients ($w_{hT,1}$, $w_{hD,1}$, $w_{hT,2}$, $w_{hD,2}$, $w_{hRec}$) for e–h injection at voxel positions 9 with stride of 5 voxels until voxel 59 for *k*, *l*, *n* in Physical Model-1 as shown in the plot legend. *Rec* in the legend refers to recombination coefficients. Readers are suggested to enlarge the figure for closer view.

We performed experimental studies with unit electron–hole charge pair injections at voxel positions 9 with stride of 5 voxels until voxel 59. The trained weights of the model for electron drift coefficients, electron and hole trapping coefficients are shown in Fig. 5a–c, respectively for different *k*, *l* and *n* in the loss function in Eq. (3). Table 1 shows the different error values of the learned model properties. The error for the electron coefficients are computed from Voxels 9 to 99 since the electrons move towards anode and the coefficients in those voxels gets trained, while for the hole coefficients, the error is computed from Voxels 1 to 59, where the hole move towards cathode. For $k=1$, $l=10^4$ and $n=10^3$, the error is minimum, as shown in Table 1. The electron trapping, detrapping and recombination coefficients fit closely to the ground truth values as shown in Fig. 5b. The drift coefficients follow the ground truth which is uniform in the material as shown in Fig. 5a. Here we consider the fact that the hole drift coefficients is one-tenth that of electron drift coefficients $(\mu _{h} = 0.1\mu _{e})$. The hole trapping, detrapping coefficients for the 2 trapping centers alongwith the recombination coefficients are shown in Fig. 5c. The holes travel from the point of injection towards the cathode which is Voxel 0 in our model. The holes get trapped inside the material due to its lower drift coefficient ($\mu _{h}$), and thus the hole coefficients are trained only in those voxels where the holes propagate.

**Table 1:** Error values for Physical Model-1. $Err(\mu _{e})$, $Err(w_{eT})$, $Err(w_{eD})$ and $Err(w_{eRec})$ are the Error values in the drift, trapping, detrapping and recombination coefficients of the electrons respectively. $Err(w_{hT,1})$, $Err(w_{hD,1})$, $Err(w_{hT,2})$, $Err(w_{hD,2})$ and $Err(w_{hRec})$ are the error values in the trapping 1, detrapping 1, trapping 2, detrapping 2 and recombination coefficients of the holes respectively. *Err*(*Mean*) is the arithmetic mean of these error values.

*k*, *l*, *n*	$Err(\mu _{e})$	$Err(w_{eT})$	$Err(w_{eD})$	$Err(w_{eRec})$	$Err(w_{hT,1})$	$Err(w_{hD,1})$	$Err(w_{hT,2})$	$Err(w_{hD,2})$	$Err(w_{hRec})$	*Err*(*Mean*)
1,$10^3$,$10^3$	2.12$\times 10^{-4}$	0.2437	0.1183	0.2567	0.0755	0.0447	0.0866	0.0574	0.1425	0.1140
1,$10^4$,$10^3$	1.55$\times 10^{-4}$	0.0700	0.0728	0.1265	0.0781	0.0447	0.0911	0.0656	0.1903	**0.0821**

Significant values are in [bold].

### Numerical experiments with Physical Model-2

**Figure 6:**
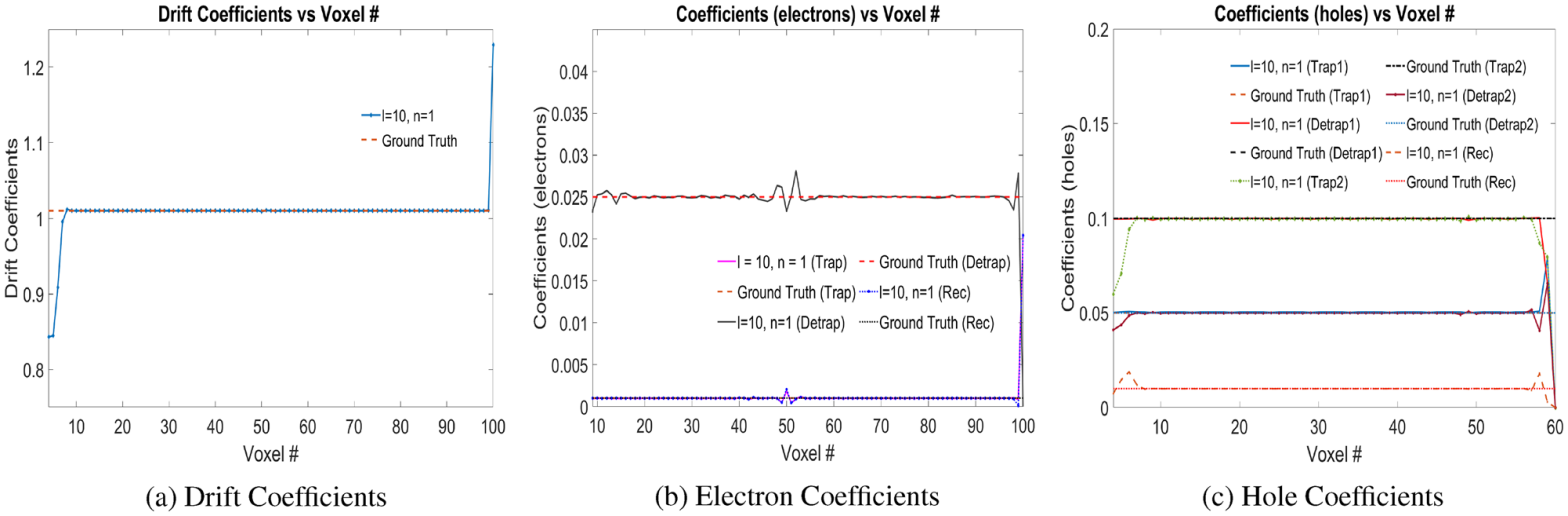
(**a**) Drift coefficients ($\mu _e$), (**b**) electron coefficients ($w_{eT}$, $w_{eD}$, $w_{eRec}$), and, (**c**) hole coefficients ($w_{hT,1}$, $w_{hD,1}$, $w_{hT,2}$, $w_{hD,2}$, $w_{hRec}$) for e–h injection at voxel positions 9 with stride of 5 voxels until voxel 59 for *l* and *n* in Physical Model-2 as shown in the plot legend. *Rec* in the legend refers to recombination coefficients. Readers are suggested to enlarge the figure for closer view.

Unit charge in terms of electron–hole pair injections at voxel positions 9 with stride of 5 voxels until voxel 59 are fed into the model in order to train the model. The trained weights of the model—drift coefficients, electron coefficients and hole coefficients are shown in Fig. 6a–c, respectively. In the weighted loss function of Eq. (4), we use the weights $l=10$, $n=1$, is based on the same ratio for the weights in the loss function of Eq. (3) used in Physical Model-1 which gives marginally smaller mean error. For the drift coefficients ($\mu _e$), the trained weights follow the ground truth as shown in Fig. 6a. The learned trapping, detrapping and recombination coefficients for the electrons match closely to the ground truth parameters as in Fig. 6b. Similarly, for holes, the trapping, detrapping coefficients for trapping centers 1 and 2, alongwith the recombination coefficients match closely to the ground truth parameters as in Fig. 6c. For the trained weights as shown in Fig. 6, the error values of drift coefficients ($\mu _e$), trapping ($w_{eT}$), detrapping ($w_{eD}$), recombination coefficients ($w_{eRec}$) for electrons are $1.6108 \times 10^{-4}$, 0.1241, 0.0240, 0.1673 respectively which are computed for voxels 9 to 99. Similarly, the error values of the trapping 1 ($w_{hT,1}$), detrapping 1 ($w_{hD,1}$), trapping 2 ($w_{hT,2}$), detrapping 2 ($w_{hD,2}$) and recombination coefficients ($w_{hRec}$) for holes are 0.0768, 0.0447, 0.1005, 0.0640, 0.2406 respectively which are computed for voxels 1 to 59. The arithmetic mean of the error of these coefficients is 0.0936.

### Numerical experiments with Physical Model-3

**Figure 7:**
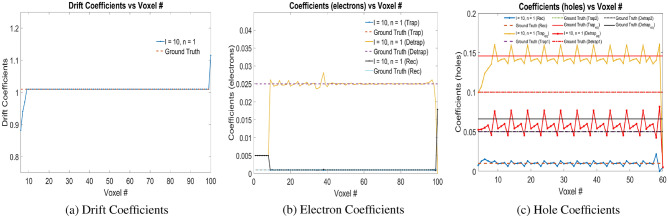
(**a**) Drift coefficients ($\mu _e$), (**b**) electron coefficients ($w_{eT,1}$, $w_{eD,1}$, $w_{eRec}$), and, (**c**) hole coefficients ($w_{hT,1}$, $w_{hT,2}$, $w_{hT,eq}$, $w_{hD,1}$, , $w_{hD,2}$, $w_{hD,eq}$, $w_{hRec}$) for e–h injection at voxel positions 9 with stride of 5 voxels until voxel 59 for *l* and *n* in Physical Model-3 as shown in the plot legend. *Rec* in the legend refers to recombination coefficients. Readers are suggested to enlarge the figure for closer view.

The electron-hole charge pairs are injected at voxel positions 9 with stride of 5 voxels until voxel 59 during training the Physical Model-3 as well. The learned physical properties of the model—drift coefficients, electron coefficients and hole coefficients are shown in Fig. 7a–c respectively. Equation (7) is used as the loss function with weights $l=10$ and $n=1$ based on the same ratio for the weights in the free and trapped electron and hole charges as in simulation experiments with Physical Model-1 and Physical Model-2. Clearly, the electron drift, trapping, detrapping and recombination coefficients follow the ground truth values. The learned recombination coefficients for the holes follow the ground truth values as well. For multiple trapping centers for holes (2 in this case), the learning based model finds the equivalent trapping center, with equivalent trapping and detrapping lifetimes following Eqs. (5) and (6) respectively.

The ground truth values of trapping 1 lifetime is 0.195 $\upmu$s and trapping 2 lifetime is 0.094 $\upmu$s. This corresponds to the probability of trapping holes in trap center 1 and 2 to be 0.05 and 0.10 respectively. The ground truth equivalent trapping lifetime ($\tau _{eq}$) is calculated using Eq. (5) to be 0.0634 $\upmu$s. The fraction of holes remaining as free holes after getting trapped in the equivalent trapping center is $N_{after} = N_{before}e^{-dt/\tau _{eq}}$. Considering time step $dt =10$ ns, $N_{after} = 0.8541N_{before}$. Thus, the fraction of holes getting trapped in the equivalent trapping center is $1-0.8541=0.1459$. Similarly, in the ground truth simulation data, we considered fraction of charge getting detrapped from trap center 1 and 2 are 0.10 and 0.05 respectively. Thus, after detrapping, the fraction of charges remaining in trapping center 1 and 2 would be 0.90 and 0.95 respectively. Considering $N_{after} = N_{before}e^{-dt/\tau _{eq}}$, with the same time step of $dt = 10$ ns, the detrapping 1 and 2 lifetimes are 94.9122 ns and 194.9573 ns respectively. Considering Eq. (6), and equivalent trapping probability as 0.1459, we compute the equivalent detrapping lifetime $\tau _{dt,eq}$ as 145.9 ns. The fraction of holes remaining after detrapping from the equivalent trapping level would be 0.9338. Thus, the fraction of charges getting detrapped from the equivalent trapping center is $1-0.9338=0.0662$. From our simulation experiments, we find that our learning based model is able to correctly identify the equivalent trapping and detrapping probabilities as shown in Fig. 7c. For the trained weights as shown in Fig. 7, the error values of drift coefficients ($\mu _e$), trapping ($w_{eT}$), detrapping ($w_{eD}$), recombination coefficients ($w_{eRec}$) for electrons are $1.32 \times 10^{-5}$, 0.0412, 0.0316, 0.0277 respectively which are computed for voxels 9 to 99. Similarly, the error values of the equivalent trapping ($w_{hT,eq}$), equivalent detrapping ($w_{hD,eq}$), and recombination coefficients ($w_{hRec}$) for holes are 0.0954, 0.1957 and 0.3378 respectively which are computed for voxels 1 to 59. The arithmetic mean of the error of these material properties is 0.1042.

### Numerical experiments with Physical Model-4

**Figure 8:**
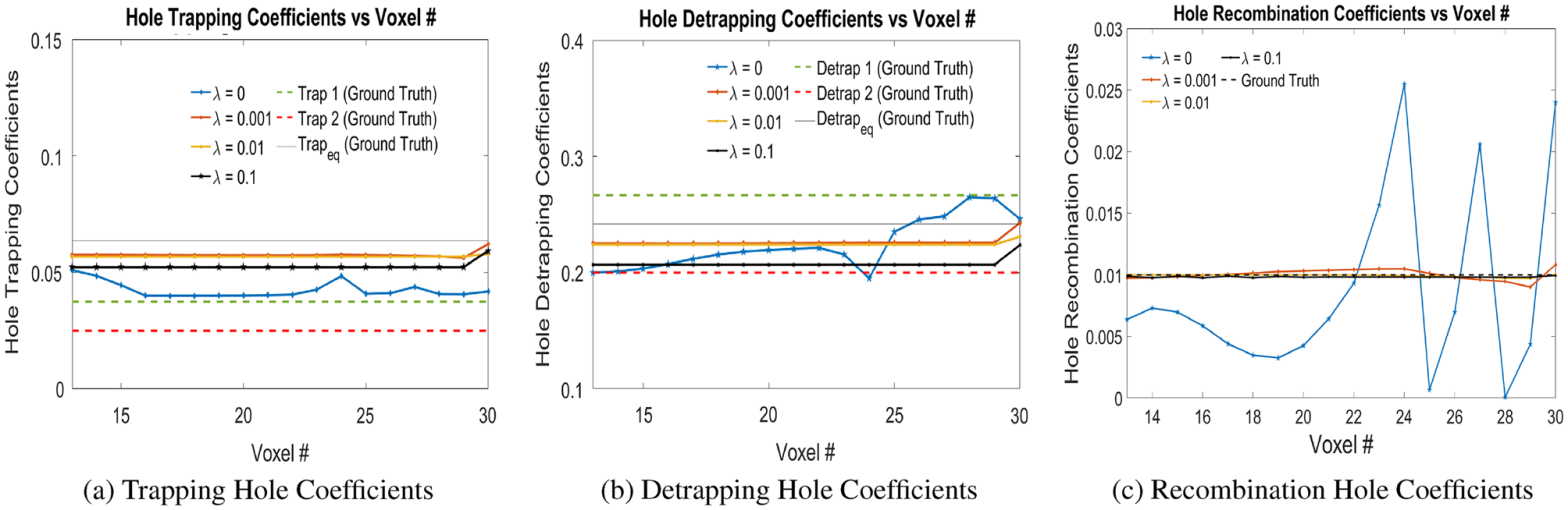
Trapping, Detrapping and Recombination Hole Coefficients for hole injection at Voxels 24, 27 and 30 in Physical Model-4 with varying $\lambda$ values as shown in the legend. Here $\lambda$ refers to $\lambda _2$ in Eq. (9). Readers are suggested to enlarge the figure for closer view.

The physical model-4 is trained with signals generated from motion due to electrons and holes separately. We show the convergence of trained hole coefficients and electrons coefficients. For holes, the physical model-4 finds out the equivalent trapping and detrapping coefficients similar to physical model-3. Figure 8 shows the hole trapping, detrapping and recombination coefficients due to electron-hole pair injection at voxel positions 24, 27 and 30 for different $\lambda _2$ in Eq. (9). During training, the trapping hole and detrapping hole weights were bounded in [0.04, 0.07], and [0.15, 0.30] respectively which are close to the actual ground truth weights. The initialization of trapping, detrapping and recombination coefficients for holes are done uniformly at 0.05, 0.2 and 0.005 respectively. This is represented as ‘bound’ in Table 3. It is seen that for $\lambda _2 = 0$, without T.V. regularization, the hole trapping, detrapping and recombination coefficients does not converge to the ground truth hole coefficients. On the other hand using the T.V. regularization improves the convergence of these coefficients to actual ground truth values. The different error values, computed for hole coefficients from Voxels 13 to 30 for varying $\lambda _2$ values are shown in Table 2. For $\lambda _2 = 0.001$ and 0.01, the hole trapping and detrapping coefficients are closer to the ground truth coefficients than for $\lambda _2 = 0.1$ and hence smaller the error. However, for recombination coefficients, the trained weights for $\lambda _2 = 0.1, 0.01$ and 0.001 are better than for $\lambda _2 = 0$. Thus, it is seen that the weights $\lambda _2 = 0.01, 0.001$ in the loss function of Eq. (9) provides better convergence for the hole coefficients.

**Table 2:** Error values for Physical Model-4 with different $\lambda _{2}$ values. $Err(w_{hT,eq})$, $Err(w_{hD,eq})$ and $Err(w_{hRec})$ are the error values in the trapping equivalent, detrapping equivalent and recombination coefficients of the holes respectively. *Err*(*Mean*) is the arithmetic mean each of these error values. Hole injections are at voxel 24, 27 and 30.

$\lambda _2$	$Err(w_{hT,eq})$	$Err(w_{hD,eq})$	$Err(w_{hRec})$	*Err*(*Mean*)
0	0.3347	0.1139	0.7515	0.4000
0.001	0.0967	0.0672	0.0427	**0.0689**
0.01	0.1072	0.0727	0.0108	**0.0636**
0.1	0.1719	0.1419	0.0175	0.1104

Significant values are in [bold].

**Table 3:** Error values for Physical Model-4 for $\lambda _{2} = 0.001, 0.01$ values. $Err(w_{hT,eq})$, $Err(w_{hD,eq})$ and $Err(w_{hRec})$ are the error values in the trapping equivalent, detrapping equivalent and recombination coefficients of the holes respectively. *Err*(*Mean*) is the arithmetic mean each of these error values. Hole injections are at voxel 24, 26 and 28.

$\lambda _2$	condition	$Err(w_{hT,eq})$	$Err(w_{hD,eq})$	$Err(w_{hRec})$	*Err*(*Mean*)
0.001	bound	0.0842	0.0548	0.0326	0.0572
0.001	far	0.1512	0.1375	0.0807	0.1231
0.001	ip1	0.0994	0.0628	0.0189	0.0604
0.001	ip2	0.0549	0.0206	0.0278	**0.0115**
0.01	bound	0.1118	0.0734	0.0161	0.0671
0.01	far	0.1884	0.1591	0.0089	0.1188
0.01	ip1	0.1154	0.0809	0.0098	0.0687
0.01	ip2	0.0341	0.0332	0.0103	**0.0259**

Significant values are in [bold].

**Figure 9:**
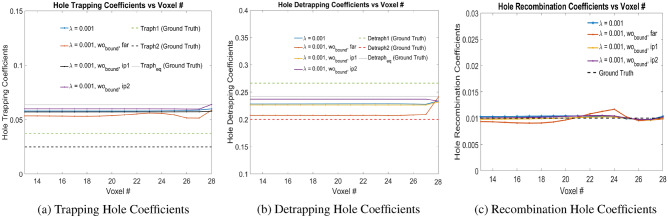
Trapping, Detrapping and Recombination Hole Coefficients for hole injection at Voxels 24, 26 and 28 in Physical Model-4 with $\lambda = 0.001$ as shown in the legend. Here $\lambda$ refers to $\lambda _2$ in Eq. (9). Readers are suggested to enlarge the figure for closer view.

**Figure 10:**
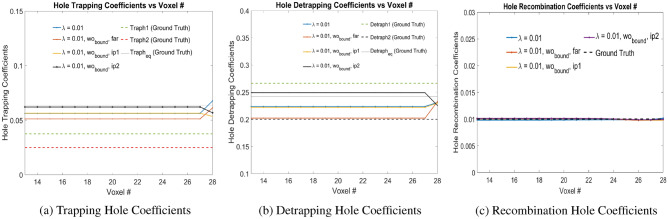
Trapping, Detrapping and Recombination Hole Coefficients for hole injection at Voxels 24, 26 and 28 in Physical Model-4 with $\lambda = 0.01$ values as shown in the legend. Here $\lambda$ refers to $\lambda _2$ in Eq. (9). Readers are suggested to enlarge the figure for closer view.

Additional simulation experiments has been done with $\lambda _2 = \{0.001, 0.01\}$ without bounds on trapping and detrapping coefficients and electron-hole pair injections at Voxels 24, 26 and 28. All the initial weights of trapping and detrapping over the voxels has been uniformly initialized as $\{0.005, 0.005\}, \{0.05, 0.2\}, \{0.07, 0.3\}$ which corresponds to ‘far’, ‘ip1’ and ‘ip2’ respectively in Figs. 9 and 10. Table 3 shows the different error values, computed for hole coefficients from Voxels 13 to 28. It is observed that bounds on the trapping and detrapping weights do not have any influence on the final trained weights of the holes. However, for $\lambda _2 = 0.001$, the hole trapping, detrapping and recombination coefficients converge more closely to the ground truth parameters. Additionally, initializing the trapping and detrapping hole weights with ‘ip2’ converges the trained weights closer to the actual ground truth parameters, which signifies better convergence of the solution to ground truth parameters with weights initialized above the ground truth parameters.

**Table 4:** Error values for Physical Model-4 with different $\lambda _{1}$ values. $Err(\mu _{e})$, $Err(w_{eT})$, $Err(w_{eD})$, $Err(w_{eRec})$ are the error values in the drift, trapping, detrapping and recombination coefficients of the electrons respectively. *Err*(*Mean*) is the arithmetic mean each of these error values.

$\lambda _1$	$Err(\mu _{e})$	$Err(w_{eT})$	$Err(w_{eD})$	$Err(w_{eRec})$	*Err*(*Mean*)
0	0.0370	0.2790	0.0723	0.4560	0.2111
0.001	0.2729	0.0544	0.0455	0.3281	0.1752
0.01	0.1970	0.0257	0.0384	0.2815	0.1311
0.1	0.0350	0.0187	0.0145	0.1868	**0.0638**

Significant values are in [bold].

**Figure 11:**
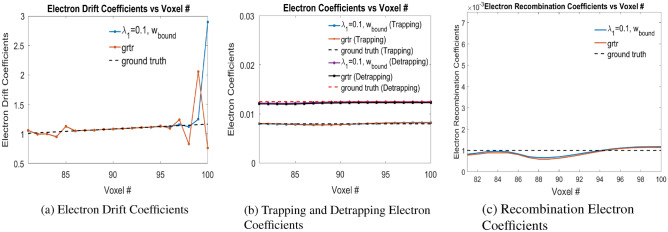
(**a**) Drift, (**b**) Trapping and Detrapping and, (**c**) Recombination Electron Coefficients for electron injection at Voxels 81, 84 and 87 in Physical Model-4 with $\lambda _{1} = 0.1$ in Eq. (9). Readers are suggested to enlarge the figure for closer view.

Similar experiments has been done with varying $\lambda _1$ in Eq. (9) with $\lambda _{1} \in \{0, 0.001, 0.01, 0.1\}$ as shown in Table 4. The electron-hole pair injections are at Voxels 81, 84 and 87. It is seen that for $\lambda _{1} = 0.1$ in Eq. (9), the mean error value has the minimum value of 0.0638. During training, the bounds on trapping, detrapping and recombination weights for electrons are bounded in [0.004, 0.012], [0.008, 0.02] and [0.0005, 0.005] respectively, while the initialization of trapping, detrapping and recombination coefficients for electrons are done uniformly at 0.012, 0.015 and 0.002 respectively. This is referred to as ‘bound’ in Table 5. Additional simulation experiments has been done with $\lambda _{1}=0.1$ without bounds on trapping, detrapping and recombination coefficients with the same electron-hole pair injections at Voxels 81, 84 and 87. The initialization of trapping, detrapping and recombination weights for electrons are done with the same values of $\{0.012, 0.015, 0.002\}$, $\{0.02, 0.02, 0.004\}$ and $\{0.005, 0.009, 0.0005\}$ which are referred as ‘same’, ‘grtr’ and ‘lt’ respectively as shown in Table 5. It is seen that for ‘grtr’ case, the mean error has the value of 0.1159 which is minimum of these three cases. Overall, the case with ‘bound’ provides us with the minimum error for the electron coefficients. Figure 11 shows the electron drift, trapping, detrapping and recombination coefficients for $\lambda =0.1$ with ‘bound’ and ’grtr’ cases. The material properties for the electrons converges very closely to the corresponding ground truth values.

**Table 5:** Error values for Physical Model-4 for $\lambda _{1} = 0.1$ at different conditions. $Err(\mu _{e})$, $Err(w_{eT})$, $Err(w_{eD})$, $Err(w_{eRec})$ are the error values in the drift, trapping, detrapping and recombination coefficients of the electrons respectively. *Err*(*Mean*) is the arithmetic mean each of these error values.

condition	$Err(\mu _{e})$	$Err(w_{eT})$	$Err(w_{eD})$	$Err(w_{eRec})$	*Err*(*Mean*)
bound	0.0350	0.0187	0.0145	0.1868	0.0638
same	0.1991	0.0203	0.0261	0.2294	0.1187
grtr	0.1930	0.0205	0.0269	0.2232	0.1159
lt	0.4140	0.0213	0.0328	0.2502	0.1796

### Comparison of the different Physical Models

The performance of the different physical models has also been evaluated with the Relative Error in %, is shown in Table 6. The mean relative error due to the electron coefficients (*Err*2(*electrons*)) are separated from that of holes (*Err*2(*holes*)) and the mean of *Err*2(*electrons*) and *Err*2(*holes*) are then computed as *Err*2(*Total*) in Table 6. For Physical Model-1, $k=1, l=10^4, n=10^3$ are used with electron–hole pair injections are voxel position 9 with stride of 5 voxels until voxel 59. For Physical Model-2, $l=10, n=1$ is used, with same electron–hole pair injections as in Physical Model-1. For Physical Model-3, $l=10, n=1$, and the electron–hole pair injections are in the same voxel position as in Physical Model-1 and 2. In Physical Model-4, the electrons and holes coefficients are trained separately. Hence, the Err2(electrons) only refers to the relative error result for the model trained for electron coefficients only. The electron injections are at voxel 81, 84 and 87 with $\lambda _{1}=0.1$ with ’bound’ condition as described in Table 5. On the other hand, Err2(holes) only refer to the relative error result for the model trained for hole coefficients only. The hole injections are at voxel 24, 26 and 28 with $\lambda _{1}=0.001$ with ’ip2’ condition as described in Table 3.

**Table 6:** Relative Error (in %) for the four different physical models.

Physical Model	*Err*2(*electrons*)	*Err*2(*holes*)	*Err*2(*Total*)
1	2.4104	2.9942	2.7023
2	2.6536	3.6098	3.1317
3	1.3443	16.0488	8.6966
4 (holes only)	$\times$	3.2256	4.1909
4 (electrons only)	5.1561	$\times$	

It is seen that for Physical Models 1 and 2, the *Err*2(*Total*) value is small. On the other hand, for Physical Model 3, the *Err*2(*holes*) are maximum. This is because in the learned model, the hole coefficients (equivalent in this case) tend to oscillate around the ground truth value. Overall, the Err2(Total) is less than $9 \%$, which shows good convergence of the RTSD material parameters to the ground truth values.

## Discussion

The learning-based approach for obtaining the detector parameters is novel for RTSD. We have developed four physical models using different amount of data for modeling the RTSD. In practice, experimentally generating data for building these learning models, such as free and trapped charges (holes and electrons) in the bulk of the material requires several experimental setups, expert know how and time. Physical learning based model have been developed step-wise using fewer data which directly relates to fewer experimental setups. Physical Model-1 uses most information regarding the system, while Physical Model-4 utilizes just signals in the learning model, which is obtained directly from the electrodes without any additional hardware requirements. We also observe that progressively using reduced or limited data in developing these models affect the characterization properties of the RTSD. While in Physical Models-1 and 2, we characterize all the properties of the RTSD, in Physical Models-3 and 4, the characterization of the RTSD is done in a single equivalent manner for multiple trapping centers (holes in this case). Thus, the Physical Model-3 and 4 is agnostic to the actual (ground truth) number of trapping centers in the RTSD. In this work, we have shown our results of a model with a 100 voxel, but our approach can be extended to models with other voxel as well. Our learning approach learns the properties of the RTSD in a fast and efficient way and can identify defects in the detector spatially and their variations over time.

In experimental results with Physical Model-3, for the hole coefficients in Fig. 7c, we observe slight fluctuations in the trained parameters around the converged value. We observe that these fluctuations gradually diminish if we continue training over additional several epochs. Additionally, in Physical Model-4, we observe that the addition of T.V. regularization to the loss function in Eq. (8), improves the solution drastically and converges the hole and electron coefficients to the ground truth parameters. However, from our numerical experiments with Physical Model-4, we observe that different weights on the T.V. regularization is required for electrons compared to the holes.

In this work, a 1D learning model of the detector trained with different amount of data is presented. The 3D learning model of the detector will follow the same principles. Moreover, in this work, the ground truth data has been simulated using a classical model in MATLAB as in our previous work^35^. In actual practice, the simulation results must be validated with actual experimental data. This experimental data can be obtained using thermoelectric emission spectroscopy, thermally stimulated current measurements, laser induced techniques and others. However, this work reduces the burden on generating experimental data and can still characterize the RTSD at higher resolution (in order of microns) than any other technique in the literature. The impact of additional noise (such as electronic noise) in the data needs to be addressed as well. Nevertheless, extending this model to work with actual experimental data in 3D detector systems is one of the future directions of work.

## Conclusion

The paper introduces novel learning-based physical models of the radiation detector using limited data, which characterizes the RTSD. The limited data dictates the requirement of fewer experimental setups and less information to train these models, which is hugely beneficial for practical wide-scale implementation. Four physical models have been demonstrated which progressively utilize fewer data and characterize the material. Based on the amount of data, the models either characterize the detector completely or in an equivalent manner. The model shows promising results which could lead the way for future developments in characterization of the RTSD with fewer experimental setups and data.

## Data Availability

The datasets used and/or analysed during the current study are available from the corresponding author on reasonable request.
